# Determining the Genetic Architecture of Reproductive Stage Drought Tolerance in Wheat Using a Correlated Trait and Correlated Marker Effect Model

**DOI:** 10.1534/g3.118.200835

**Published:** 2018-12-12

**Authors:** Rudy Dolferus, Saravanan Thavamanikumar, Harriet Sangma, Sue Kleven, Xiaomei Wallace, Kerrie Forrest, Gregory Rebetzke, Matthew Hayden, Lauren Borg, Alison Smith, Brian Cullis

**Affiliations:** * CSIRO Agriculture and Food, GPO Box 1700, Canberra ACT 2601, Australia; † Department of Environment and Primary Industry, AgriBioSciences, La Trobe R&D Park, Bundoora, VIC 3083, Australia; ‡ National Institute for Applied Statistics Research Australia (NIASRA), School of Mathematics & Applied Statistics, Faculty of Engineering & Information Sciences, University of Wollongong NSW 2522, Australia

**Keywords:** osmotic stress/drought tolerance/wheat/grain number/QTL/mixed model/WGAIM

## Abstract

Water stress during reproductive growth is a major yield constraint for wheat (*Triticum aestivum* L). We previously established a controlled environment drought tolerance phenotyping method targeting the young microspore stage of pollen development. This method eliminates stress avoidance based on flowering time. We substituted soil drought treatments by a reproducible osmotic stress treatment using hydroponics and NaCl as osmolyte. Salt exclusion in hexaploid wheat avoids salt toxicity, causing osmotic stress. A Cranbrook x Halberd doubled haploid (DH) population was phenotyped by scoring spike grain numbers of unstressed (SGNCon) and osmotically stressed (SGNTrt) plants. Grain number data were analyzed using a linear mixed model (LMM) that included genetic correlations between the SGNCon and SGNTrt traits. Viewing this as a genetic regression of SGNTrt on SGNCon allowed derivation of a stress tolerance trait (SGNTol). Importantly, and by definition of the trait, the genetic effects for SGNTol are statistically independent of those for SGNCon. Thus they represent non-pleiotropic effects associated with the stress treatment that are independent of the control treatment. QTL mapping was conducted using a whole genome approach in which the LMM included all traits and all markers simultaneously. The marker effects within chromosomes were assumed to follow a spatial correlation model. This resulted in smooth marker profiles that could be used to identify positions of putative QTL. The most influential QTL were located on chromosome 5A for SGNTol (126cM; contributed by Halberd), 5A for SGNCon (141cM; Cranbrook) and 2A for SGNTrt (116cM; Cranbrook). Sensitive and tolerant population tail lines all showed matching soil drought tolerance phenotypes, confirming that osmotic stress is a valid surrogate screening method.

Drought stress is one of the most common causes of yield loss in wheat. About 50% of the global wheat growing area (230 million hectares) is affected by drought, with average yields ranging between an estimated 10–50% of the theoretical irrigated potential (Pfeiffer *et al.* 2005; Morris *et al.* 1991). In Europe, climate change causing drought and heat events is considered the main reason behind the stagnation of yield growth rates in wheat (Brisson *et al.* 2010). Droughts occur frequently in Australia, causing severe wheat yield losses (Richards *et al.* 2014). Changing rainfall patterns are likely to result in more frequent occurrences of droughts in the future. Drought tolerance is therefore an essential trait that needs incorporating in cereals to secure yield stability (Powell *et al.* 2012; Semenov and Halford 2009).

Progress in drought tolerance breeding has been slow for several reasons. Response of plants to drought stress remains poorly understood and involves complex gene networks. Selection in a field environment is notoriously variable; occurrence of drought stress, its severity and timing during plant development and interference of other environmental factors (*e.g.*, heat) are beyond control. QTL mapping attempts in wheat have so far led to identification of a multitude of genetic loci with poor phenotypic contributions (Bennett *et al.* 2012; Fleury *et al.* 2010; Foulkes *et al.* 2007; Nakhforoosh *et al.* 2015; Acuña-Galindo *et al.* 2015; Bonneau *et al.* 2013). A major impediment to improving drought tolerance in wheat remains accuracy of phenotyping. Many drought traits focus on improving water use efficiency and vegetative growth. These traits are aimed at boosting crop yields by increasing harvest index, stem carbohydrate levels, tiller number, plant height, water use and transpiration efficiency, carbon isotope discrimination and root depth (Blum 2005; Collins *et al.* 2008; Salekdeh *et al.* 2009; Fleury *et al.* 2010; Rebetzke *et al.* 2013). Although these traits managed to gradually improve wheat productivity in water-challenged environments (Richards *et al.* 2014), they did not contribute significantly to drought tolerance *per se* (Condon *et al.* 2004; Blum 2005; Saradadevi *et al.* 2017a, b). There are very few drought-induced tolerance traits in wheat. Stay-green is based on maintaining vegetative growth by delaying leaf senescence, which improves vegetative stage biomass accumulation and drought tolerance. But stay-green does not guarantee higher grain yields when droughts coincide with reproductive development (Gregersen *et al.* 2013; Thomas and Ougham 2014). Because water stress conditions most often occur during the reproductive stage, drought-tolerance traits that target reproductive development are also required. Osmotic adjustment is a drought-induced tolerance trait that also protects reproductive development. Osmotic adjustment is strongly correlated with drought tolerance in wheat (Morgan 1983; Morgan and Condon 1986; Morgan 2000), but the trait is difficult to use for large scale germplasm screening.

Loss in grain number is generally considered the main contributor to drought-induced yield losses (Fischer 1973; Saini and Aspinall 1981; Savin and Slafer 1991; Fischer 1993; González *et al.* 2003; Sreenivasulu and Schnurbusch 2012). Grain number is affected during the earlier stages of reproductive development, ranging from early spike differentiation to meiosis and early gametophyte development (Dolferus *et al.* 2011; Dolferus 2014). The young microspore stage (YM) of pollen development is particularly sensitive to abiotic stresses. In an autogamous plant like wheat maintaining pollen fertility is essential to maintain grain number (Ji *et al.* 2010; Dolferus *et al.* 2013). The aim of this paper was to establish selection for maintenance of pollen fertility and grain number as a reproductive stage drought tolerance trait. To focus on maintenance of grain number rather than pure grain number and yield, we made several changes to the phenotyping protocol and used a novel QTL mapping strategy. First, controlled environment phenotyping was used to control occurrence, duration and severity of drought conditions. Second, stress conditions were always imposed at the YM stage of pollen development and irrespective of flowering time, enabling us to eliminate avoidance or escape reactions (Shavrukov *et al.* 2017). Third, osmotic stress was used as a surrogate stress treatment to replace notoriously variable soil drought conditions. This made it possible to reduce variability in duration and severity of stress treatments. This was achieved by using a hydroponics facility and stressing plants using NaCl as osmoticum (Munns and James 2003; Munns *et al.* 2010). Fourthly, plants were grown to determine both unstressed and stressed spike grain numbers, allowing us to avoid intrinsic variation in spike grain number. Finally, we had to establish a QTL mapping procedure that allowed us to map genetic loci for stress tolerance or the capacity to maintain grain number.

The investigation of plant variety tolerance to stress factors via experiments with stressed and unstressed treatment regimes is common. The data from such experiments can be viewed as comprising two traits, namely the trait under stress conditions and the trait under control conditions. Typically there is a positive genetic correlation between the traits which has a regression interpretation in the sense that, on average, varieties that are superior under the control treatment tend also to be superior under the stress treatment. This makes unraveling the genetic architecture of “tolerance” a complex task. Certainly, QTL mapping of the stress trait alone or the difference between the stress and control traits does not target tolerance. We have tackled this issue using a linear mixed model (LMM) analysis that includes a correlation between the marker additive effects for the stressed and unstressed traits. The implied genetic regression allows derivation of a tolerance trait that reflects the non-pleiotropic effects associated with the stress treatment that are independent of the control treatment. Our derivation is analogous to that proposed in Lemerle *et al.* (2006) but is based on a LMM that includes marker score data. Our model with marker data are similar to the WGAIM model of Verbyla *et al.* (2007) in that it is a whole genome approach, so all markers are included simultaneously. We have extended their approach to encompass the bivariate modeling of the stress and control treatments, and, in particular the associated genetic regressions which define tolerance. Furthermore, Verbyla *et al.* (2007) assume that the marker effects are independent whereas we assume the effects (for a given treatment) to be correlated, with the correlation being a decaying function of the distance between the markers. This results in smooth marker profiles (plots of predicted marker effects against marker position) that can be used to identify important genomic regions and thence putative QTL. This avoids the multiple testing issues inherent in most standard QTL mapping approaches.

## Materials and Methods

### Plant material, soil drought treatments and linkage map construction

The wheat varieties used in this study were previously tested for soil drought tolerance at the sensitive YM stage: drought-sensitive Sundor and Cranbrook, and drought-tolerant Halberd and AUS30604 (Ji *et al.* 2010). Soil drought stress treatments were completely as described before (Ji *et al.* 2010; Dolferus *et al.* 2013). Plants were grown in trays (15 plants per tray). Water withholding and tagging of tillers was started 1-2 days prior to reaching the YM stage and stress treatments were carried out for 3 days. Plants were then re-watered and grown until maturity.

The Cranbrook × Halberd DH population consists of 166 DH lines (Kammholz *et al.* 2001), all of which were genotyped using a 90K SNP chip containing gene-associated SNPs that provide dense coverage of the wheat genome (Wang *et al.* 2014). The SNP markers were complemented with the genotypes of the photoperiod gene *PPD-D1*, vernalisation gene *VRN-A1* and the semi-dwarf locus *Rht1*; these genes are polymorphic between the Cranbrook and Halberd population parents (Eagles *et al.* 2010, 2014, 2009). The linkage map was constructed using ASMap (Taylor and Butler 2014), a program which wraps MSTMap (Wu *et al.* 2008) in R (R-Development-Core-Team 2014). The resulting linkage groups were assigned to wheat chromosomes based on 90K SNP chip data (Wang *et al.* 2014). A detailed report of the linkage map construction is contained in (Cullis *et al.* 2016).

### Molecular and biochemical measurements of ABA metabolism

Wheat spike RNA extraction (YM stage) and real-time PCR gene expression studies (*TaZEP1*) were completely as described previously (Ji *et al.* 2011). Gene expression studies were carried out for three repeats of spike RNA samples collected from 3 individual plants. ABA measurements in wheat spikes were carried out using a Phytodetek competitive ELISA kit (Agdia) as described by Ji *et al.* (2011). ABA measurements were repeated three times using a mix of YM stage spikes from three individual plants.

### Osmotic stress phenotyping and experimental design

**Figure 1:**
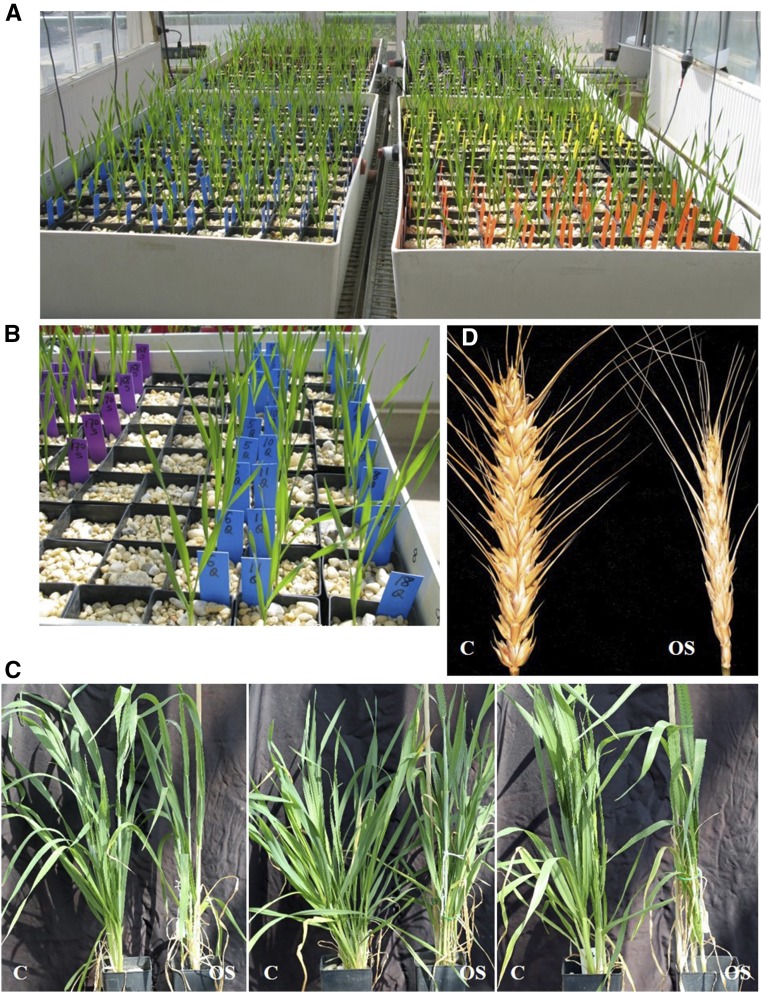
Hydroponics setup for growing the wheat DH population and osmotic stress treatments. (A) DH lines (three pots per line) were grown in large tanks that were periodically flushed with Hoagland medium. (B) Plants were grown in pots filled with fine quartz gravel. (C) Appearance of plants after five days of stress treatment in Hoagland medium containing NaCl as osmoticum. Control (left, C) and osmotic stress (right, OS) treated plants of three population lines showed weak signs of water stress. (D) At maturity, spike grain number is determined for each of the DH lines.

The data considered in this paper come from a glasshouse experiment involving the factorial combination of the 166 DH lines and parental checks with two treatments, namely a control and osmotic stress treatment (to be described later). Three seeds per line and checks were imbibed for 2h in water and surface sterilized for one minute with a 0.14% Thiram (Bayer CropScience, USA) fungicide solution. The seeds were then blotted dry to remove excess Thiram and transferred to a wet filter paper placed in flat-bottomed 12-well microwell plates for pre-germination. Pre-germination occurred in the dark and individual seedlings were then transplanted into square pots (65mm wide × 160mm high) filled with fine quartz gravel (Figure 1 A, B). Individual seedlings were transplanted per pot, using three pots for each line (one untreated control check and two pots for osmotic stress treatments).

Pots were placed into hydroponic tanks on benches within a glasshouse. Each tank accommodated a maximum of 144 pots arranged in a 16 row × 9 column array. The DH lines were grouped according to their average flowering time calculated from different field growing seasons at Yanco and Narrabri Experimental Stations, New South Wales. This resulted in five so-called YM groups (very quick, quick, moderate, slow and very slow). The number of lines allocated to each flowering group grown in each run is presented in Supplementary Table S1. All lines within a maturity group were located together in the glasshouse in so-called maturity blocks. Whenever blocks were adjacent within a tank at least one row was left vacant between them to avoid shading between lines within different YM groups. The lines were randomized to triples of row-adjacent pots, here-after called main plots, within their respective maturity block. The osmotic stress was randomly assigned to two of these pots within the main plot and the remaining pot was then assigned to the control treatment (non-stressed). The layout of pots and allocation of DH lines to main plots for one of the tanks in the glasshouse is given in Table 1.

**Table 1:** Schematic layout of experimental design for tank 2 in osmotic stress phenotyping. The tank comprised 144 pots arranged in a 16 row by 9 column array. Each cell in the figure represents a triplet of pots (across 3 columns within the row) and is termed a main plot. The first part of the label in each cell is the DH line that was allocated to all 3 pots within the main plot. Two of the pots within each main plot were allocated to the stress treatment and the remaining pot was allocated to the control treatment (see Supplementary Fig. S1). The numbers of tillers measured for each treatment in the main plot is given as the final part of the label (*e.g.*, 2, 3 means there were measurements for 2 tillers from the control pot and 3 from the treated pots) Genetic clones of DH lines are shown using equality symbols (*e.g.*, CH#135 (=151) means line CH#135 was genetically identical to line CH#151)

		Columns		
		1-3	4-6	7-9
**Rows**	**1**	CH#55 (=43,56,57): 2, 3	CH#49: 2, 4	CH#46: 1, 3
	**2**	CH#60: 2, 3	CH#57 (=43,55,57): 3, 5	CH#56 (=43,55,57): 2, 3
	**3**	CH#81 (=80): 2, 2	CH#74: 3, 3	CH#71: 4, 6
	**4**	CH#86: 2, 4	CH#83: 3, 4	CH#82: 2, 4
	**5**	CH#89: 2, 2	CH#88: 3, 5	CH#87: 2, 3
	**6**	CH#93: 2, 3	CH#91: 3, 2	CH#90: 2, 4
	**7**	CH#98: 3, 2	CH#97: 3, 7	CH#94: 1, 2
	**8**	CH#102: 2, 2	CH#101: 2, 4	CH#100: 2, 3
	**9**	CH#108: 2, 4	CH#107: 1, 2	CH#106: 1, 4
	**10**	CH#121: 2, 4	CH#119: 4, 4	CH#112: 2, 3
	**11**	CH#128: 3, 5	CH#127: 2, 5	CH#126: 2, 5
	**12**	CH#135 (=151): 2, 2	CH#134: 4, 5	CH#133: 2, 4
	**13**	CH#145 (=149): 1, 1	CH#141: 3, 6	CH#138: 2, 4
	**14**	CH#154: 2, 5		CH#147: 2, 3
	**15**	CH#165: 2, 2	CH#160: 4, 3	CH#155: 2, 2
	**16**			CH#169: 2, 4

The tanks were periodically flushed with water for 3 min periods, and then drained. The height of the irrigation water was adjusted to keep seedling roots moist. This irrigation cycle was repeated every 30 min and after one week the water was replaced by quarter-strength Hoagland’s medium (Hoagland and Arnon 1938). After a further two weeks this was increased to full strength. The pH of the tanks was monitored weekly and maintained at 6.5-7.0. The level of the nutrient storage tanks was monitored weekly and topped up with de-ionized water to compensate for evaporation. Nutrient solutions were refreshed every two weeks to control algal growth.

The determination of the stress-sensitive YM stage was based on auricle distance (AD) measurements as described before (Ji *et al.* 2010; Dolferus *et al.* 2013). The AD to reach the YM stage varies between the different lines of the DH population (ranges between +3 and +8), but this variation is offset by the fact that different florets of a wheat spike and spikes on different tillers are not synchronized in flowering. Meiosis starts in the middle and proceeds toward the top and base of the spike and the two basal (outer) florets of each spikelet are ahead in development compared to the third and fourth (inner) florets. Because of the length of the stress treatment, the sensitive stage had to be anticipated with the aim to maximize the number of florets going through meiosis during the stress treatment (Dolferus *et al.* 2013). We therefore determined an AD interval (-1 to +11; roughly corresponding to Zadok scores 39 to 47) to capture a maximal amount of florets on different tillers per plant going through meiosis during the stress treatment. This interval captures most of the meiosis events in the spike and is well before occurrence of anthesis (Zadok 62-68). The tillers at the right stage within the pots assigned to the osmotic stress treatments were tagged and these pots were then placed in separate smaller tanks in order to apply the treatment (Supplementary Fig. S1; untreated control plants remain in the larger tank). To avoid shock effect, the treatment was applied by increasing the salt concentration in incremental steps in four separate smaller tanks; the Hoagland medium in each of these tanks was supplemented with 100, 150, 200 and 250mM NaCl respectively. To compensate for lower calcium uptake in the presence of NaCl, salt solutions were supplemented with Ca^2+^ to adjust the Na^+^:Ca^2+^ ratio to 15:1 (Munns and James 2003; Hoagland and Arnon 1938). The root system of plants was irrigated for 3 min with the media and then allowed to drain. This process was repeated every 30 min. Plants were kept at each of the first three levels for one day and for two days at the 250mM highest concentration. Total duration of the stress treatment was 5 days. The plants were then stepped back to normal Hoagland medium along the same salt gradient over one day (1h per step) and returned into their respective original positions in the large tank until maturity (Supplementary Fig. S1). The date the stress treatment was applied was recorded for each pot assigned to the stress treatment and was used as the time-to- YM-stage (TYM) trait for QTL mapping. At maturity, spikes were harvested individually and spike grain numbers were recorded. The summary of the number of spikes per treatment is presented in Supplementary Table S2. The number of spikes per treatment is variable for the DH lines and there were generally more spikes for the stress treatment (two pots per DH line were used).

The entire experiment was repeated in the same glasshouse and the data from the two runs was combined for analysis. There were therefore two distinct sources of experimental error to be accommodated in the analysis, namely within and between run error. The experimental design was non-standard and complicated by the fact that the same randomization of lines to main plots was used for each run, the maturity blocks were located in the same positions in each run and the allocation of treatments to pots within main plots was not recorded. In terms of within run error we note that the design for each run was strictly un-replicated for DH lines as the experimental units for DH lines are the main plots and there was only a single main plot for each DH line. Valid replication of lines and treatment by line combinations within runs was enabled through the genetic analysis in which genetic clones were identified (see later). A total of 10 clones was identified, with one clone comprising four DH lines; one comprising three DH lines and eight comprising 2 lines. Thus, for example, the clone comprising four DH lines appeared in four main plots and thence had four replicates within each run. Table 1 shows that tank 2 included three of the four replicate main plots for this clone. The identification of genetic clones meant that there was minimal partial replication (Cullis *et al.*, 2006) of the lines and line by treatment combinations for the estimation of within run error. However, this was offset by the fact that the analysis also included between run error in the form of interaction effects of runs with lines and treatments. This is arguably a more important source of error in terms of generalizing the results, as it represents replication of the entire experimental protocol, including the preparation of the saline solution used for the stress treatment and exposure to a new set of conditions.

### Statistical Analysis

#### Baseline analysis:

The data for all spikes for both treatments were analyzed together using a single LMM in which both genetic and non-genetic effects were included. The latter were included to encapsulate the plot structure of the experimental design as described in the previous section. The genetic effects related to the factorial combinations of the 143 DH lines that had genotyping data (see later) and the two treatments. We may regard the two sets of effects as representing two traits, namely spike grain number in the presence (SGNTrt) and absence (SGNCon) of the osmotic stress treatment. Formally, we let $u_{gi+}$ and $u_{gi-}$ be the true (total) genetic effect for the $i^{th}$ DH line (i=1…143) for the stress and control treatments respectively. These effects were accommodated in the model using a bivariate structure which includes a separate variance (that is, a genetic variance) for each set of effects, denoted $\sigma_{\;g+}^{2}$ and $\sigma_{\;g-}^{2}$, for the stress and control treatments respectively, and a covariance between them, denoted $\sigma_{g\pm }$. Thus the bivariate model allows for a genetic correlation between the two traits and this is given by:

$$\rho_{g\pm }=\;\sigma_{g\pm }/\sqrt{\sigma_{\;g+}^{2}\sigma_{\;g-}^{2}\;}$$

Correlation between two traits can also be viewed as a regression of one trait on the other and we exploit this in order to define a third trait of interest, namely tolerance (also see Lemerle *et al.* 2006). In our context we consider the regression of the true genetic effects associated with the stress treatment on the effects associated with the control treatment, which can be written for the $i^{th}$ DH line as:

$$u_{gi+}=\beta_{g}u_{gi-}+\delta_{gi}$$

where $\beta_{g}$ is the slope of the regression and is given by $\sigma_{g\pm }\;/\sigma_{\;g-}^{2}$. If the genetic correlation between SGNTrt and SGNCon is positive, then so too is the slope. In this case, the regression reflects the fact that, on average, DH lines with higher genetic effects under the control treatment also have higher genetic effects under the stress treatment. Departures from this average response are represented by $\delta_{gi}$ since this is the deviation (or residual) from the regression line for the $i^{th}$ DH line. These deviations therefore define the third trait of tolerance (SGNTol). A DH line that has tolerance would have a large positive value for $\delta_{gi}$, reflecting the fact that, when subjected to the osmotic stress treatment, this line has a higher spike grain number than would be expected given its genetic effect under the control treatment. It is important to note that the genetic effects for SGNTol and SGNCon are statistically independent, that is, cor ($\delta_{gi},\;u_{gi-}$) = 0.

Note that a shifted power transformation was applied to the spike grain number data prior to analysis in order to better satisfy the assumption of normality. The treatment structure for TYM did not involve both a control and stress treatment so the genetic effects were simplified accordingly to include only the main effects of DH lines.

All linear mixed models in this paper were fitted using ASReml-R (Butler *et al.* 2009) which provides residual maximum likelihood (REML) estimates of variance parameters, empirical best linear unbiased predictions (EBLUPs) of random effects and empirical best linear unbiased estimates (EBLUEs) of fixed effects.

#### Analysis including marker information:

The identification of putative QTL for each of the three traits of interest, namely SGNTrt, SGNCon and SGNTol, involved an extension of the baseline model to include marker score information for r=1,383 markers. This resulted in the partitioning of the total genetic effects into additive and residual genetic effects. Thus the true total genetic effect for the $i^{th}$ DH line for the stress and control treatments can be written as:

$$u_{gi+}=\overset{r}{\underset{j=1}{\sum }}M_{ij}\alpha_{j+}+u_{ei+}\;\;\&\;\;u_{gi-}=\overset{r}{\underset{j=1}{\sum }}M_{ij}\alpha_{j-}+u_{ei-}\;$$

Where $M_{ij}$ is the score for the $j^{th}\;$marker for the $i^{th}$ DH line and $\alpha_{j+}$ is the effect for the $j^{th}$ marker for the stress treatment. Marker scores reflect the two possible genotypes (Cranbrook, C; Halberd, H) and are coded as 1 or -1, respectively (for non-imputed data). This coding means that if the effect $\alpha_{j+}$ is positive, then the phenotypic value is increased for DH lines with a C type at this locus, and decreased for DH lines with an H type. The reverse is true when the effect $\alpha_{j+}$ is negative. The sum, $u_{ai+}=\;\overset{r}{\underset{j=1}{\sum }}M_{ij}\alpha_{j+}$, represents the (marker) additive genetic effect for the $i^{th}$ DH line for the stress treatment and has an associated additive variance denoted by $\sigma_{\;a+}^{2}$. The residual genetic effect,$\;u_{ei+}$, represents the genetic effect not accounted for by the markers for the stress treatment, and has associated variance $\sigma_{\;e+}^{2}$. The effects for the control treatment are defined in an analogous manner. The regression that was used to define the tolerance trait in the baseline model is generalized here in the sense that there is a separate regression for the additive and non-additive effects. In particular the regression for the additive effects is given by:

$$u_{ai+}=\beta_{a}u_{ai-}+\delta_{ai}$$

where $\beta_{a}\;$is the slope of the regression and is given by $\sigma_{a\pm }/\sigma_{\;a-}^{2}$. It can be shown that this also indicates there is a regression for the marker effects and it is given by:

$$\alpha_{j+}=\beta_{a}\alpha_{j-}+\delta_{aj}$$

The effects $\delta_{aj}\left(j=1\ldots 1,383\right)$ are deviations from the marker effect regression so represent marker effects for tolerance. The marker effects for SGNTol and SGNCon are statistically independent, that is, cor ($\delta_{aj},\;\alpha_{j-})\;$= 0. The marker effects for SGNTol therefore capture the non-pleiotropic effects associated with the stress treatment that are independent of the control treatment.

Note that all 1,383 markers are included simultaneously in the LMM so this is a whole genome approach. The marker effects (for a given treatment) are assumed to be correlated, with the correlation being a decaying function of the distance (in centi-Morgans, cM) between the markers. We use a model often associated with spatial modeling, namely a differentiable Matern model, and assume the effects to be correlated within linkage groups but uncorrelated between linkage groups. The reader is referred to Hughes (2018) for a full account of the correlated marker effect modeling.

#### Identification of putative QTL:

The use of the differentiable Matern covariance function results in smooth marker profiles (plots of EBLUPs of marker effects against marker position) for each of the three traits. We use these profiles to define regions, which are sets of consecutive markers with effects of the same sign. The contribution to the trait for the region is defined to be the total of the effects across all markers in the region. Genomic regions of interest are therefore regions with large (absolute) values for the total effect, and the putative QTL is defined to be located at the “peak” marker, that is, the marker with the largest (absolute) effect in the region. As a confirmatory step, we took the top markers defined in this way and conducted a (fixed effects) backward elimination procedure. The number of markers used in this step was chosen in such a way that together, these markers accounted for 100% of the marker additive variance for the trait concerned. Backward elimination continued until the percentage marker additive variance accounted for by the markers fell below a threshold of 99%. The final (remaining) set of markers was summarized using *p*-values and LOD scores in line with standard approaches.

### Data availability

The SNP genotyping files used to construct the linkage map, as well as the phenotyping data used in the QTL analysis are available in de-identified format at the Figshare archive: G3mark3.xlsx, G3pheno3.xlsx. Supplemental material available at Figshare: https://doi.org/10.25387/g3.6959360.

## Results

### Osmotic stress as a surrogate screening method for drought stress

The parental lines of the DH population used in this study, Cranbrook (sensitive) and Halberd (tolerant) essentially showed the same stress response phenotype under osmotic stress and soil drought conditions (Supplementary Fig. S2). We also investigated the response of the wheat lines in terms of ABA accumulation (Supplementary Fig. S3). ABA accumulates in the spikes of sensitive line Sundor following both osmotic stress treatment but not in tolerant line AUS30604. The response of ABA accumulation to osmotic stress is similar to that we previously observed for soil drought stress (Ji *et al.* 2011). Compared to soil drought, osmotic stress treatments resulted in weaker signs of leaf wilting and leaf senescence was observed for the oldest leaves at the base of the plants (Figure 1 C). After the 5-day treatment, there were no obvious signs of leaf tip necrosis, which indicates that there is no toxic effect of the salt treatment. Similar to soil drought stress, osmotic stress resulted in high levels of sterility and reduction in grain number for drought-sensitive lines (Figure 1 D). The wheat lines used in this study behave in a very similar way to osmotic stress and soil drought treatment, suggesting that osmotic stress can be used as surrogate treatment for drought stress.

### SNP genotyping and genetic map construction

Genotyping the Cranbrook × Halberd DH lines using the 90K Illumina SNP platform (Wang *et al.* 2014) yielded 16,228 polymorphic SNP markers. We complemented the SNP markers with three anchored phenological markers that were known to be polymorphic between the population parents: *VRN-A1*, *PPD-D1* and *Rht1*. The SNP markers were reduced to a set of 1,383 non-redundant markers by removing co-located markers, markers with high segregation distortion and markers with more than 20% missing data. Remaining markers were then assigned to linkage groups according to previously described principles (Wang *et al.* 2014; Taylor 2015). Nine genotypes with an exceedingly high number of crossover events were also removed from the map data. Ten genetic clones were identified showing more than 99.5% of matched marker alleles. These matching DH lines were merged to provide a final population of 143 “real” genotypes. The 90K SNP consensus map (Wang *et al.* 2014) was used as a reference during the establishment of the final Cranbrook x Halberd map. The final Cranbrook× Halberd genetic map was constructed using 1,383 markers for143 genotypes (Supplementary Fig. S4). These markers were distributed across 21 linkage groups with an average of 66 markers per linkage group. In general, the D genome had less marker density compared to the A and B genomes. The total map length was 3,866.7cM with an average length of 184.1cM per linkage group.

### Mapping of time-to-young-microspore (TYM) QTL

**Figure 2:**
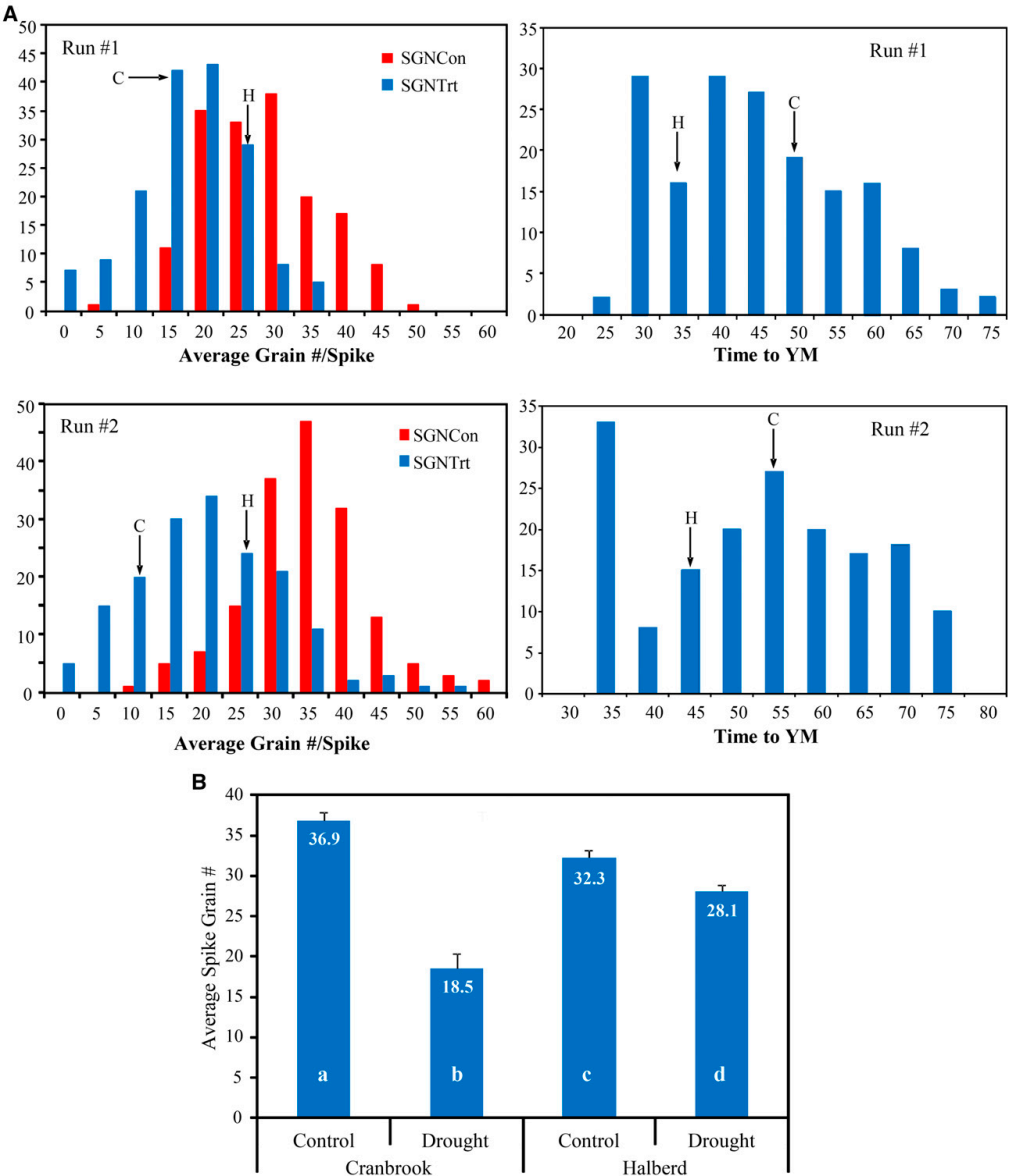
Osmotic stress phenotyping of the Cranbrook × Halberd DH population lines using the hydroponics system. (A) Distribution curves for SGNCon and SGNTrt for the two biological repeat phenotyping runs of the Cranbrook × Halberd DH population (left), as well as the distribution in TYM in both phenotyping runs. The position of the parental phenotypes (H = Halberd; C = Cranbrook) is indicated by the arrows. (B) Spike grain number under unstressed control (SGNCon) and osmotic drought stress conditions (SGNTrt) for the parental lines of the DH population, Cranbrook and Halberd. SGNCon is highest in Cranbrook, but the tolerant line Halberd is able to maintain a higher spike grain number after osmotic stress treatment (SGNTrt). Numbers in the bars indicate average spike grain numbers and bars labeled with different letters differ significantly (*P* > 0.05).

**Figure 3:**
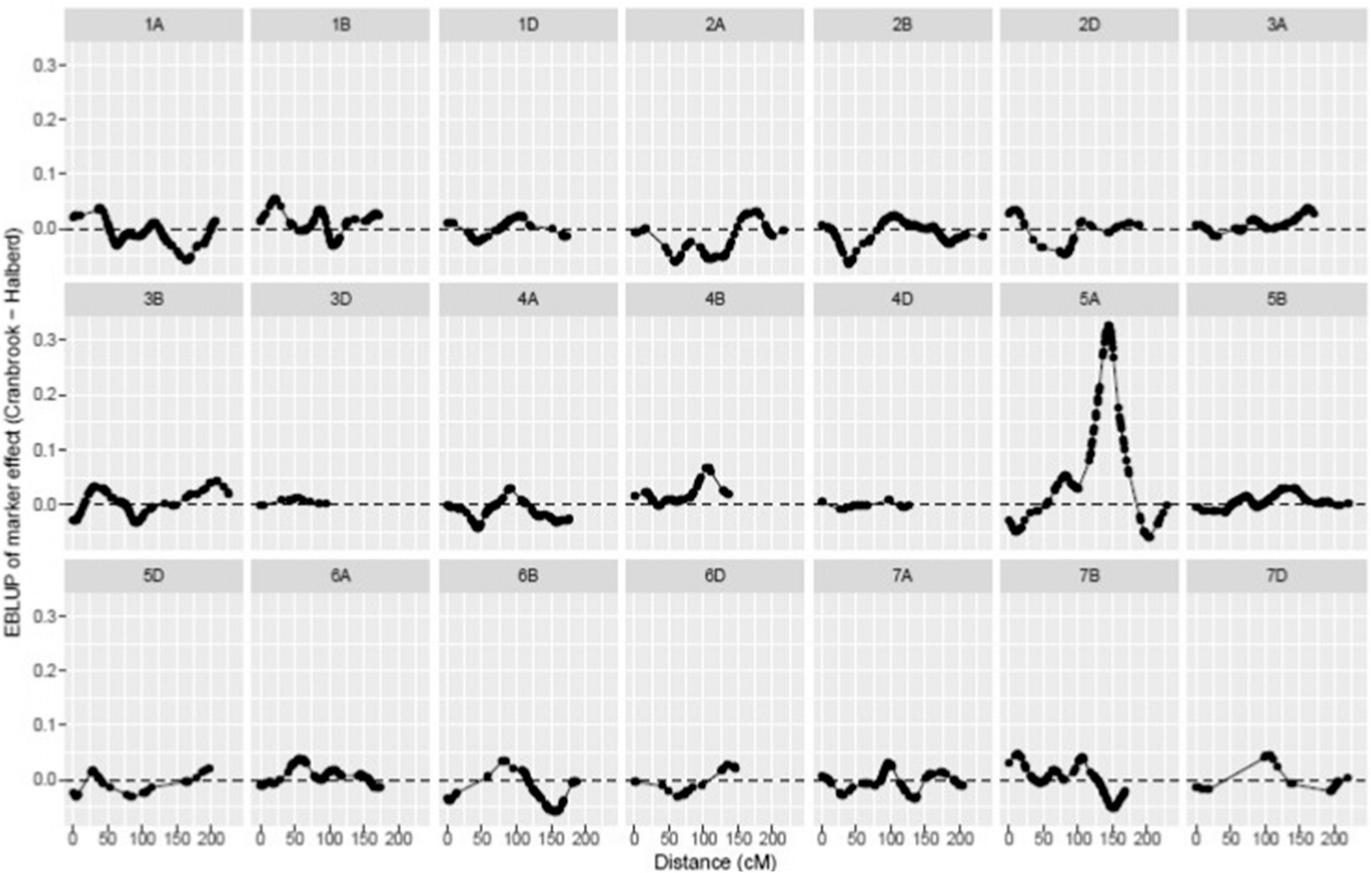
EBLUPs of marker effects for TYM plotted against genetic distance (cM) for individual linkage groups.

Because flowering time can be an escape mechanism for stress conditions, we mapped QTL for the time required to reach the young microspore stage (TYM). The flowering time (FT) in wheat is normally scored at anthesis (Zadoks 62-68). We found that there is a strong correlation between TYM and FT for the lines of this population (*r* = 0.75-0.85). There was considerable variation in time to reach the YM stage (TYM) in the DH population (Figure 2 A). Halberd takes on average 39 days to reach YM stage, while TYM for Cranbrook is 51 days. The TYM was recorded for each DH line at the start of the stress treatment. The baseline analysis (that is, without marker data) revealed a high accuracy of 0.97 for this trait. Accuracy was calculated as the average of the correlation between the true and predicted DH line effects (Mrode 2005) and we note that squared accuracy may be thought of in a similar manner to line mean heritability. Inclusion of the marker data resulted in the profiles (graphs of marker EBLUPs plotted against the genetic distance of the linkage groups) as given in Figure 3. The entire set of 1,383 markers accounted for 68.9% of the genetic variance in TYM. The top ten regions identified on Figure 3 are listed in Table 2, together with the results of the backward elimination procedure. Figure 3 and Table 2 show a clear dominant region on chromosome 5A and the marker at the peak of the region corresponds to the vernalisation gene *VRN-A1* at position 144cM. Backward elimination resulted in seven markers and confirmed the *VRN-A1* position to be the most significant, with a LOD score of 74 (Table 2). The drought-tolerant parental allele (Halberd) at this position was associated with early TYM. The association of *VRN-A1* with flowering time in wheat was not unexpected and has been described earlier (Trevaskis *et al.* 2007).

**Table 2:** Influential genomic regions for TYM. Ten regions with the largest total effects, and that contain more than 30 markers, are listed in descending order of absolute total effect. The regional information comprises the linkage group (LG), the total effect, the contributing parent and the number of markers. Note that the impact of the region on the phenotype is obtained by multiplying the total effect by the marker scores (1 for Cranbrook and -1 for Halberd). The name and distance correspond to the marker with the maximum absolute effect within the region. The final two columns are the *p*-value and LOD score for the markers that remained after the backward elimination step (that commenced with all ten markers listed in the Table)

LG	Total effect	Contribution	No. Markers	Marker	Distance (cM)	*p*-value	LOD
5A	8.555	Cranbrook	63	VRN-A1	144	<0.0001	73.66
2A	−1.941	Halberd	50	IWB72377	59	0.0062	1.63
6B	−1.292	Halberd	39	IWB42940	157	0.0035	1.85
7B	−0.988	Halberd	31	IWB2239	150		
1A	−0.965	Halberd	33	IWA3378	167	0.0109	1.41
4B	0.843	Cranbrook	39	IWA27	104	0.0003	2.85
4A	−0.690	Halberd	36	IWB31311	44	0.0505	0.83
2B	−0.631	Halberd	35	IWB28651	185	0.0193	1.19
1A	−0.631	Halberd	39	IWB28415	64		
5B	0.629	Cranbrook	47	IWB36364	141		

### Mapping of spike grain number QTL

**Figure 4:**
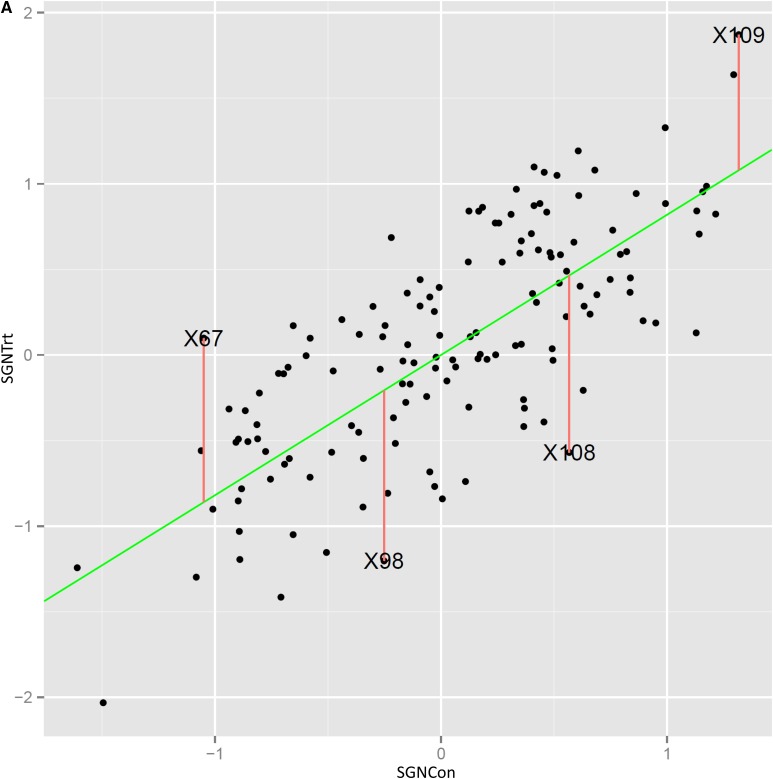

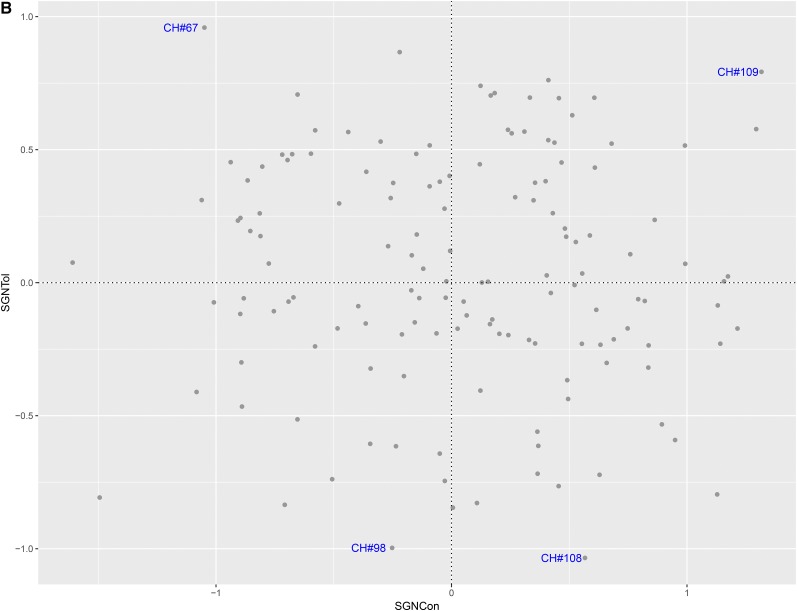
A: EBLUPs of additive genetic effects for DH lines for SGNTrt plotted against those for SGNCon. The genetic regression line is shown. The four labeled points correspond to DH lines with the largest deviations from the regression line (X = CH). B: EBLUPs of additive genetic effects for DH lines for SGNTol plotted against those for SGNCon. (see next page)

The average spike grain number of the two parents of the DH population under unstressed conditions differs, with Cranbrook spikes producing an average of 36.9 grains per spike compared to 32.3 in the drought-tolerant parent Halberd (*P* < 0.01; Figure 2 B). Following drought treatment, Halberd maintained on average 28.1 grains/spike (87%) compared to 18.5 (50%) in the sensitive parent Cranbrook (Figure 2 B). The aim of the mapping strategy was to identify the genetic capacity to maintain spike grain number under osmotic stress conditions as stress tolerance trait. To do this, we recorded spike grain numbers of unstressed control plants and stressed plants to compare stress-induced changes in spike grain number. This strategy also allowed us to neutralize the segregation in spike grain number between the DH lines of the population and focus on the genetic ability to maintain spike grain number. The SGNTol stress tolerance trait (see Materials and Methods) was designed to achieve this. The Cranbrook × Halberd DH population was phenotyped twice and individual spikes were harvested at maturity for determination of spike grain number under unstressed (SGNCon) and stressed (SGNTrt) conditions. The result of each phenotyping run is shown in Figure 2 A. The SGNCon and SGNTrt traits are correlated, but for sensitive lines SGNTrt values will be significantly lower than SGNCon. The spike grain number distribution for both control and osmotic stress treatment (SGNCon, SGNTrt) shows significant variation in the DH population and there is transgressive segregation in both directions with respect to the population parents. The distribution of the stress phenotypes indicates that the osmotic stress treatment was carried out at the right level of severity, providing phenotypic information for lines with higher tolerance and lower sensitivity to osmotic stress (Figure 2 A). The REML estimates of (total) genetic variance from the baseline analysis were 0.290 and 1.777 for SGNCon and SGNTrt, respectively. The average accuracy of these traits was 0.66 and 0.73. The estimated genetic correlation between the two traits was 0.90 and the fact that this is not unity indicates the existence of the tolerance trait (SGNTol, see Materials and Methods). The average accuracy of SGNTol (0.37) was relatively low, however, and this reflects the difficulty in targeting tolerance. The REML estimates of additive and residual genetic variances and correlations from the model that includes marker data are given in Table 3. Importantly, the marker additive effects have accounted for a large percentage of the genetic effects for SGNTol and SGNCon (100% and 88% respectively), but contribute a much smaller amount for SGNTrt (42%). It should be noted that the figure of 100% for SGNTol should be viewed with some caution as the reliability of this trait was low leading to reduced confidence in the estimates of sources of variation. The genetic correlation between the additive genetic effects for SGNCon and SGNTrt was estimated at 0.69 which indicates that, although there is general agreement between the two sets of effects, there is differential tolerance to osmotic stress. This can also be seen in Figure 4 A in which the EBLUPs of the additive genetic effects for SGNTrt are plotted against those for SGNCon. The regression line implicit in the correlation structure is drawn in Figure 4 A and has a slope given by ${\hat{\beta }}_{a}=0.82$. The additive effects for the trait of tolerance (SGNTol) are defined in terms of the vertical deviations from the regression line in Figure 4 A. DH lines that lie well above the regression line have additive effects for spike grain number under stress treatment that exceed expectation, given their additive effects under the control treatment, so may be thought of as having above average additive effects for tolerance. Examples are DH lines CH#109 and CH#67. The opposite is true for DH lines that lie well below the regression line. Examples are DH lines CH#108 and CH#98. Figure 4. B shows the EBLUPs of the additive genetic effects for SGNTol (that is, the vertical deviations from the regression line in Figure 4 A) plotted against those for SGNCon. This clearly empirically demonstrates the lack of correlation (statistical independence) between the two sets of effects. The comparison of Figure 4 A and Figure 4 B illustrates how the selection of superior lines using SGNTrt will largely reflect selection for SGNCon, whereas selection using SGNTol is unrelated to selection for SGNCon. In terms of QTL mapping this translates to the ability of the SGNTol trait to target non-pleiotropic effects for tolerance.

**Table 3:** REML estimates of marker additive and residual genetic variance for the traits of SGNCon, SGNTrt and SGNTol. *p*-values are given for the test of zero marker additive variance for each trait; marker additive variance is given as a percentage of total genetic variance. Final row gives the REML estimates of the genetic correlations between the traits of SGNCon and SGNTrt

	Additive	*p*-value	Residual	% Additive
SGNCon variance	0.6039	<0.0001	0.0825	88
SGNTrt variance	0.8547	0.002	1.186	42
SGNTol variance	0.4487	0.0097	0	100
SGNCon, SGNTrt correlation	0.69		>0.99	

### Identification of putative QTL

**Figure 5:**
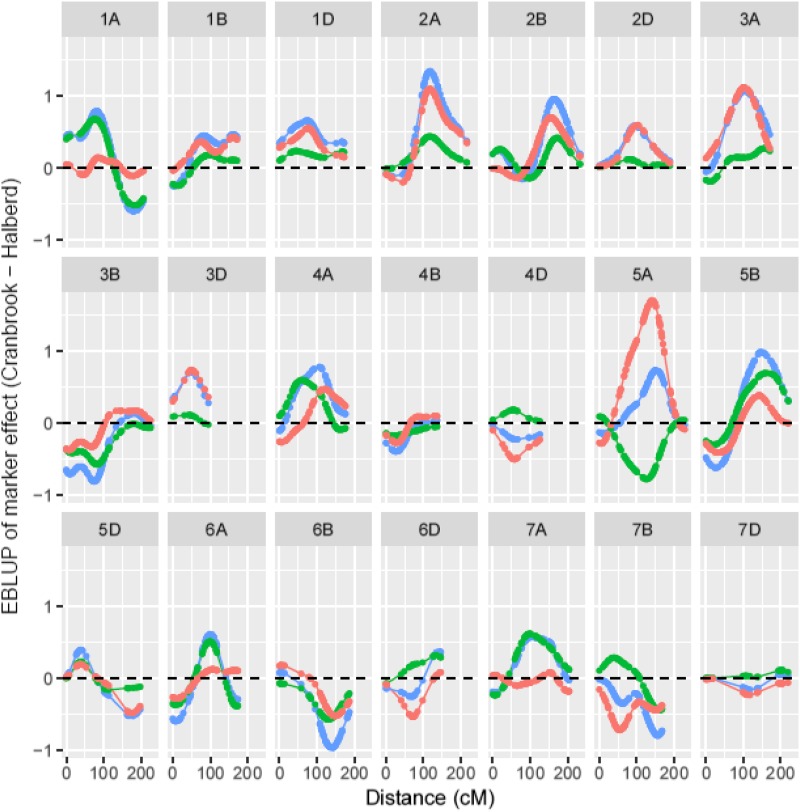
EBLUPs of marker effects for all three spike grain number traits: SGNCon (red), SGNTol (green) and SGNTrt (blue) plotted against genetic distance (cM) for individual linkage groups.

**Figure 6:**
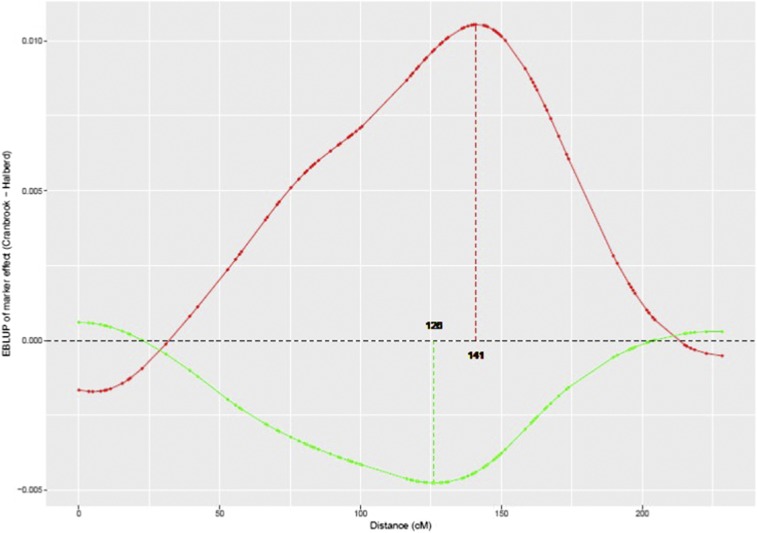
EBLUPs of marker effects for SGNCon (red) and SGNTol (green) plotted against genetic distance (cM) for chromosome 5A. Position of peak markers is explicitly shown using dashed vertical lines.

The marker profiles (EBLUPs of the marker effects plotted against marker location) for all three spike grain number traits are shown together in Figure 5. The top ten regions identified on Figure 5 for SGNCon are listed in Table 4. The most influential regions are located on chromosomes 5A, 3A and 2A. Their favorable effect is contributed by the Cranbrook parent which has higher spike grain number than Halberd. The backward elimination procedure resulted in five markers which were identical to those identified as the top five regions (Table 4). The most significant QTL was located on 5A at position 141cM, which is in proximity to the *VRN-A1* locus at position 144.4cM. The top ten regions identified on Figure 5 for SGNTol are listed in Table 5. The backward elimination procedure resulted in five markers which related to five within the top six regions, the omission being the region on 3B. The most significant QTL was located on 5A at position 126cM and was contributed by the tolerant Halberd parent. The top 15 regions identified on Figure 5 for SGNTrt are listed in Table 6. Note that 15 rather than ten regions were required for the backward elimination procedure for this trait as the top ten did not account for 100% of the marker additive genetic variance. The backward elimination procedure resulted in six markers which did not show good agreement with the ranking of the regions, apart from the top 2 marker positions. This discrepancy, and the need for more markers initially, is likely due to the low percentage of genetic variance (42%, see Table 3) accounted for by (all) the markers. This in turn illustrates the dangers in using the SGNTrt stress treatment trait to map QTL for “tolerance”. The most significant QTL was located on 2A at position 116cM and was contributed by Cranbrook. The putative QTL on 5A for SGNTol and SGNCon map relatively close to each other, at 126cM and 141cM, respectively. This can be seen in Figure 5 but more explicitly in Figure 6. However, their different parental contributions suggests that the main stress tolerance and spike grain number traits on chromosome 5A are contributing different genetic effects.

**Table 4:** Influential genomic regions for SGNCon. Ten regions with the largest total effects, and that contain more than 30 markers, are listed in descending order of absolute total effect. The regional information comprises the linkage group (LG), the total effect, the contributing parent and the number of markers. Note that the impact of the region on the phenotype is obtained by multiplying the total effect by the marker scores (1 for Cranbrook and -1 for Halberd). The name and distance correspond to the marker with the maximum absolute effect within the region. The final two columns are the *p*-value and LOD score for the markers that remained after the backward elimination step (that commenced with all ten markers listed in the Table)

LG	Total effect	Contribution	No. Markers	Marker	Distance (cM)	*p*-value	LOD
5A	0.516	Cranbrook	76	IWB55564	141	<0.0001	5.93
3A	0.286	Cranbrook	67	IWB30485	102	0.0056	1.67
2A	0.271	Cranbrook	58	IWB48486	116	0.0077	1.54
7B	−0.254	Halberd	87	IWB40092	54	0.0142	1.31
2B	0.228	Cranbrook	81	IWB26048	154	0.0079	1.53
1B	0.131	Cranbrook	74	IWB66475	161		
1D	0.126	Cranbrook	53	IWB7914	77		
6B	−0.116	Halberd	48	IWB21973	149		
3B	−0.113	Halberd	69	IWB23456	7		
4A	0.089	Cranbrook	47	IWB41760	122		

**Table 5:** Influential genomic regions for SGNTol. Ten regions with the largest total effects, and that contain more than 30 markers, are listed in descending order of absolute total effect. The regional information comprises the linkage group (LG), the total effect, the contributing parent and the number of markers. Note that the impact of the region on the phenotype is obtained by multiplying the total effect by the marker scores (1 for Cranbrook and -1 for Halberd). The name and distance correspond to the marker with the maximum absolute effect within the region. The final two columns are the p-value and LOD score for the markers that remained after the backward elimination step (that commenced with all ten markers listed in the Table)

LG	Total effect	Contribution	No. Markers	Marker	Distance (cM)	*p*-value	LOD
5A	−0.238	Halberd	76	IWA5668	126	0.0092	1.47
3B	−0.22	Halberd	97	IWB59720	83		
1A	0.22	Cranbrook	69	IWB47804	76	0.0158	1.26
5B	0.195	Cranbrook	71	IWB31506	163	0.0145	1.3
7A	0.171	Cranbrook	65	IWA3557	99	0.0571	0.79
4A	0.148	Cranbrook	58	IWA7058	60	0.0573	0.78
6B	−0.143	Halberd	60	IWB35399	131		
2A	0.113	Cranbrook	67	IWB48486	116		
2B	0.109	Cranbrook	59	IWB39236	172		
1A	−0.086	Halberd	37	IWB34474	181		

**Table 6:** Influential genomic regions for SGNTrt. Fifteen regions with the largest total effects, and that contain more than 30 markers, are listed in descending order of absolute total effect. The regional information comprises the linkage group (LG), the total effect, the contributing parent and the number of markers. Note that the impact of the region on the phenotype is obtained by multiplying the total effect by the marker scores (1 for Cranbrook and -1 for Halberd). The name and distance correspond to the marker with the maximum absolute effect within the region. The final two columns are the *p*-value and LOD score for the markers that remained after the backward elimination step (that commenced with all fifteen markers listed in the Table)

LG	Total effect	Contribution	No. Markers	Marker	Distance (cM)	*p*-value	LOD
2A	0.332	Cranbrook	63	IWB48486	116	0.0002	2.96
3B	−0.303	Halberd	76	IWB48116	75	0.0129	1.34
2B	0.287	Cranbrook	72	IWB67729	164		
3A	0.282	Cranbrook	62	IWA5982	103		
5B	0.246	Cranbrook	64	IWB71751	147	0.0217	1.15
1A	0.241	Cranbrook	72	IWB39366	80		
6B	−0.233	Halberd	51	IWB33580	142	0.0334	0.98
7B	−0.224	Halberd	87	IWB62272	157		
4A	0.196	Cranbrook	71	IWB31578	107	0.0089	1.49
5A	0.186	Cranbrook	77	IWB34320	151		
1D	0.168	Cranbrook	53	IWB19029	73		
7A	0.156	Cranbrook	60	IWB59436	102		
1B	0.153	Cranbrook	66	IWB66475	161		
5B	−0.102	Halberd	39	IWB4020	26	0.023	1.12
1A	−0.093	Halberd	34	IWB34474	181		

### Osmotic stress-tolerant lines are drought-tolerant

**Figure 7:**
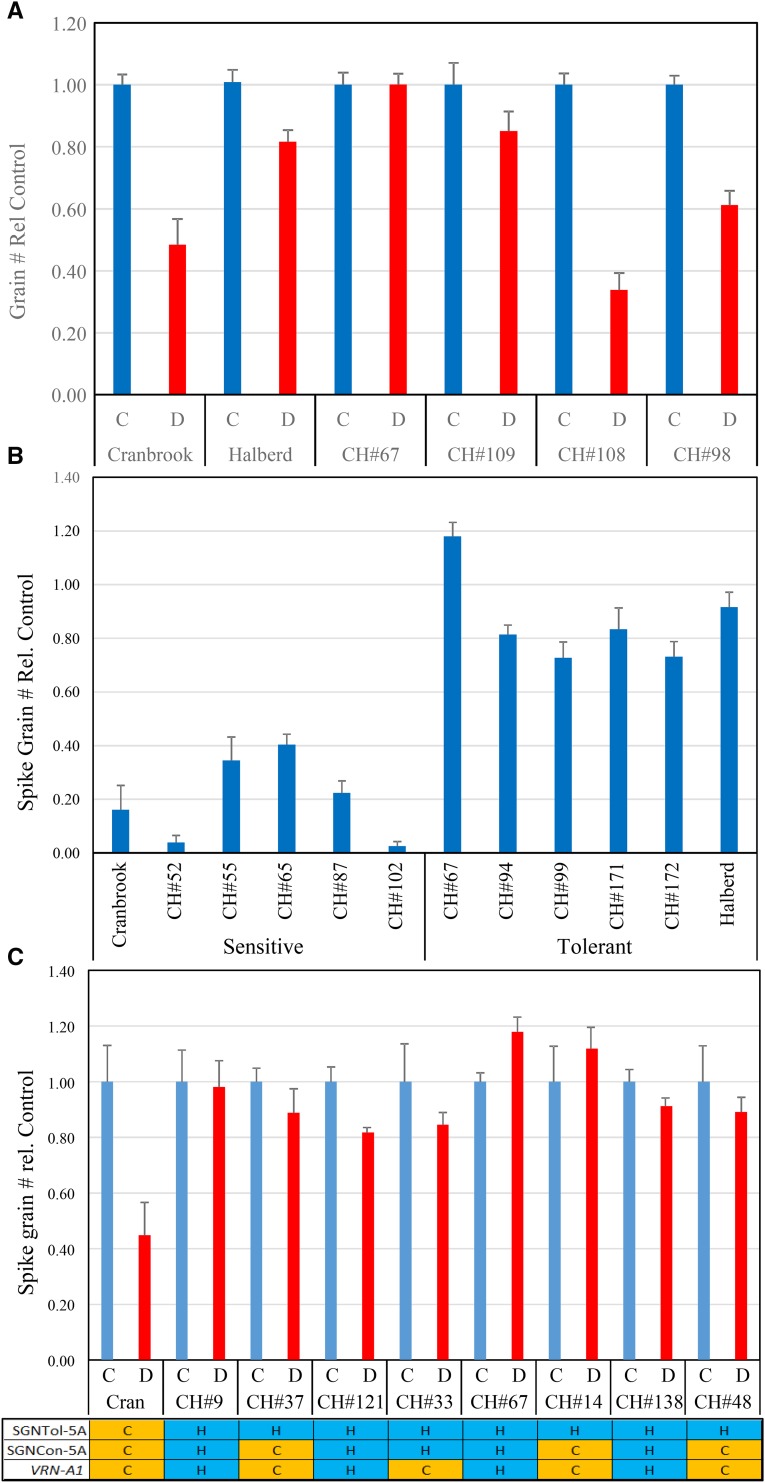
Confirmation of soil drought tolerance of tested osmotic stress DH lines. (A) Soil drought tolerance testing of the 4 lines indicated in the linear regression plot of Fig. 4. The results confirm that the two lines with the largest positive deviation from the regression line (CH#67 and CH#109) are drought-tolerant, while the two lines with the largest negative deviation from the regression line (CH#98 and CH#108) are sensitive to drought stress. (B) Drought-tolerance testing for some of the most osmotic stress-tolerant and sensitive tail lines of the Cranbrook x Halberd population confirms that their drought- and osmotic-stress tolerance phenotype are matching. (C) Drought-tolerance testing of population lines showing recombination between the Halberd tolerance allele for SGNTol-5A (H) and the closely linked SGNCon-5A and *VRN-A1* (TYM-5A) alleles (Halberd, H; Cranbrook, C). The results show that lines with the SGNTol-5A tolerant allele from Halberd are drought-tolerant irrespective of the SGNCon-5A and *VRN-A1* alleles (H or C). The sterility data are averages of spike grain number data of 20-30 spikes and two biological repeats. The error bars represent the standard errors.

We tested the soil drought tolerance of the 4 lines indicated in Figure 4. The two lines with largest deviation (SGNTol) above the regression line, CH#67 and CH#109, both proved to be better able to maintain spike grain number under soil drought conditions (Figure 7 A). The two lines with largest deviation below the regression line, CH#98 and CH#108, both proved to be more sensitive to soil drought conditions (Figure 7 A). We also tested five osmotic stress tolerant and five osmotic stress sensitive tail lines for soil drought tolerance (Figure 7 B). These lines were chosen because they were consistently tolerant or sensitive in the two biological repeats of the two osmotic stress phenotyping runs used for the QTL analysis. All these lines have the SGNTol-5A allele from tolerant parent Halberd. The results indicate that the soil drought tolerance phenotype matched the osmotic tolerance phenotype for all the lines tested (Figure 7 B). Osmotic stress tolerance phenotyping is therefore a suitable surrogate screening method for drought tolerance in wheat.

The favorable Halberd allele for SGNTol-5A (144.4cM) is normally closely linked to the SGNCon-5A and *VRN-A1* loci. The distance to the SGNCon-5A (141cM) and *VRN-A1* loci is 18.4cM and the observed number of recombinants between the SGNTol peak and *VRN-A1* in the DH population is 13.4%. To demonstrate that the SGNTol-5A locus contributes to stress tolerance and not the linked SGNCon-5A and *VRN-A1* loci, we tested recombinant lines of the DH population where the Halberd allele of SGNTol-5A is linked to Cranbrook alleles of SGNCon-5A and *VRN-A1*. All but one of the lines we tested (CH#33) also showed recombination between the peak marker of SGNCon-5A (IWB55564) and the SGNTol-5A Halberd allele (Figure 7 C). All lines with the Halberd SGNTol-5A allele were drought-tolerant irrespective of the origin of the SGNCon-5A and *VRN-A1* alleles, indicating that the stress tolerance phenotype contributed by the Halberd SGNTol-5A allele (Figure 7 C). The SGNTol-5A locus affects drought tolerance independently from the SGNCon-5A and *VRN-A1* loci.

## Discussion

Soil drought conditions are variable and hard to control, even in controlled environments (Passioura 2007, 2012). Controlling stress severity for different lines of a mapping population that flower at different times is therefore hard to achieve. Particularly, because our phenotyping method aims to apply drought stress at the YM stage, irrespective of flowering time. To solve this problem we resorted to hydroponics using osmotic stress as a replacement for soil drought stress. Polyethylene glycol (PEG) and mannitol have previously been used as non-ionic osmolytes to apply osmotic stress but these osmotica are not resistant to bacterial growth and can have toxic side effects (PEG). Because of the salt exclusion capacity of hexaploid wheat we used NaCl as osmoticum (Schachtman *et al.* 1992; Munns 2002; Munns *et al.* 2010). The response of wheat to salinity occurs in two phases: a rapid osmotic stress phase that inhibits growth of new leaves and a slower toxic ionic phase (Munns *et al.* 1995). Salt exclusion by roots allows wheat to keep Na^+^ and Cl^−^ concentrations below toxic levels in the aerial plant parts, in particular in growing cells and reproductive organs (Munns and Rawson 1999; Munns 2002). Germination of hexaploid wheat can resist high NaCl concentrations (400mM), while concentrations from 150 to 250mM NaCl can discriminate genotypic differences in stomatal conductance and leaf elongation rate. Accumulation of salt in wheat leaves and especially reproductive structures was shown to remain below toxic levels (Munns and Rawson 1999; Munns 2002; Rahnama *et al.* 2010). The osmotic stress treatment we used was of short duration and did not result in obvious tissue necrosis, suggesting that plant material did not experience the toxic ionic phase of salt stress. Osmotic stress treatment in hydroponics mimicked the effect of soil drought treatments and the contrasting wheat lines used in this study performed in the same way under both treatments.

Both drought stress and osmotic stress affect the plant’s water balance. Under water stress conditions plants can either reduce stomatal conductance to limit water loss, or they can leave stomata open and maintain their water balance through osmotic adjustment and increased water uptake by the roots (Blum 2009, 2015). Accumulation of ABA activates stomatal closure and an associated repression of photosynthesis and metabolic activity (Blum 2015; Schachtman and Goodger 2008). Osmotic adjustment does not require ABA accumulation; stomata remain open for continued photosynthesis and root growth and phloem transport is continued to sustain turgor pressure (Morgan 1984; Morgan and King 1984; Cushman 2001; Osakabe *et al.* 2013). Wheat lines with reproductive drought tolerance such as Halberd prevent ABA accumulation in the spike while sensitive lines do accumulate ABA (Ji *et al.* 2011). ABA measurements and expression studies of the ABA biosynthetic gene *TaZEP1* indicate that osmotic stress has the same effect as drought stress on ABA accumulation. This suggests that the tolerant and sensitive Halberd and Cranbrook lines used in this study differ in their potential to osmotically adjust to maintain turgor pressure and reproductive development. Osmotic adjustment is highly correlated with drought tolerance in wheat (Morgan 1984, 2000; Živčák *et al.* 2009), so osmotic stress phenotyping is a suitable method for identifying QTL for water stress tolerance in wheat.

The difficulty in identifying genetic loci for drought tolerance is interference of stress avoidance mechanisms associated with plant phenology. In addition, many drought traits are directly or indirectly focused on grain yield, making it difficult to identify true drought-tolerance traits (Blum 2005; Fleury *et al.* 2010; Rollins *et al.* 2013). The vernalization gene *VRN-A1* on chromosome 5A is an important wheat adaptability trait with pleiotropic effects on grain yield (Snape *et al.* 1985). *VRN-A1* frequently co-localises with QTL for abiotic stress “tolerance” and ABA accumulation when selection is based on grain number (Snape *et al.* 1985; Quarrie *et al.* 1994; Bálint *et al.* 2009; Pinto *et al.* 2010; Iehisa *et al.* 2014; Wang *et al.* 2016; Onyemaobi *et al.* 2018). We therefore designed a QTL mapping approach aimed at identifying drought tolerance loci (that is, capacity to maintain spike grain number) rather than grain yield QTL. QTL analysis was carried out for three traits: SGNCon and SGNTrt are spike grain number traits for unstressed and stressed conditions respectively, and SGNTol is a tolerance trait derived from the two spike grain number traits. SGNTol is constructed in such a way that, unlike SGNTrt, the genetic (and marker) effects for SGNTol are statistically independent of (uncorrelated with) those for SGNCon. Thus they can be used to target non-pleiotropic tolerance effects. The mapping results show that there is very little overlap between the position of the peaks and flanking markers for the mapped chromosome regions for these three traits. The main SGNTol QTL is located on chromosome 5A (126cM), but the main SGNCon and TYM QTL also map to different positions on chromosome 5A, but with different parental contributions. The TYM flowering time QTL has *VRN-A1* as peak marker, while the SGNCon-5A peak is at 141cM, a position very closely linked to *VRN-A1* marker (144cM). The SGNTrt-5A peak marker is located at 151cM. Both spike grain number traits are contributed by the stress-sensitive parent Cranbrook. In contrast, the SGNTol-5A stress tolerance trait is contributed by the tolerant parent Halberd. Lines of the mapping population with the Halberd allele of the SGNTol-5A QTL show higher soil drought tolerance and the osmotic stress tolerance phenotype matches the drought tolerance phenotype for all lines tested. These results suggest that the drought tolerance trait used in this study is able to identify previously unidentified regions of the wheat genome. We identified some population lines that showed recombination between the Halberd allele for SGNTol-5A and the linked SGNCon-5A and *VRN-A1* loci (TYM-5A) and found that the SGNTol-5A QTL is responsible for the drought-tolerance phenotype, irrespective of the SGNCon-5A and *VRN-A1* alleles.

In conclusion, controlled environment phenotyping using a surrogate osmotic stress tolerance trait and a novel QTL mapping approach have enabled us to identify some novel genomic regions that enable wheat to maintain pollen fertility and spike grain number under reproductive stage drought conditions. Fine-mapping is currently in progress for the main SGNTol QTL on chromosome 5A. The fact that drought tolerance is controlled by ten genomic regions (five QTL) with positive contributions of both parents suggests that the SGNTol trait is controlled by a complex gene network. A genomic selection strategy using molecular markers from all SGNTol regions identified in this study may offer a better way forward to improve reproductive stage drought tolerance in wheat.
